# Editorial: Autophagy-Mediated Cell Survival and Death in Disease Progression and Treatment

**DOI:** 10.3389/fcell.2022.916347

**Published:** 2022-07-04

**Authors:** Yongqiang Chen, Yongchao Zhao, Paras Kumar Mishra

**Affiliations:** ^1^ CancerCare Manitoba Research Institute, CancerCare Manitoba, University of Manitoba, Winnipeg, MB, Canada; ^2^ Department of Hepatobiliary and Pancreatic Surgery, Zhejiang Provincial Key Laboratory of Pancreatic Disease, The First Affiliated Hospital, Institute of Translational Medicine, Cancer Center, Zhejiang University School of Medicine, Zhejiang University, Hangzhou, China; ^3^ Department of Cellular and Integrative Physiology, University of Nebraska Medical Center, Omaha, NE, United States

**Keywords:** autophagy, ATG4B, ATG4D, cell death, eicosapentaenoic acid (EPA), TSSC4 (tumor suppressing subtransferable candidate 4), ULK1, breast cancer

## Introduction

### Rationale for This Research Topic

**FIGURE 1:**
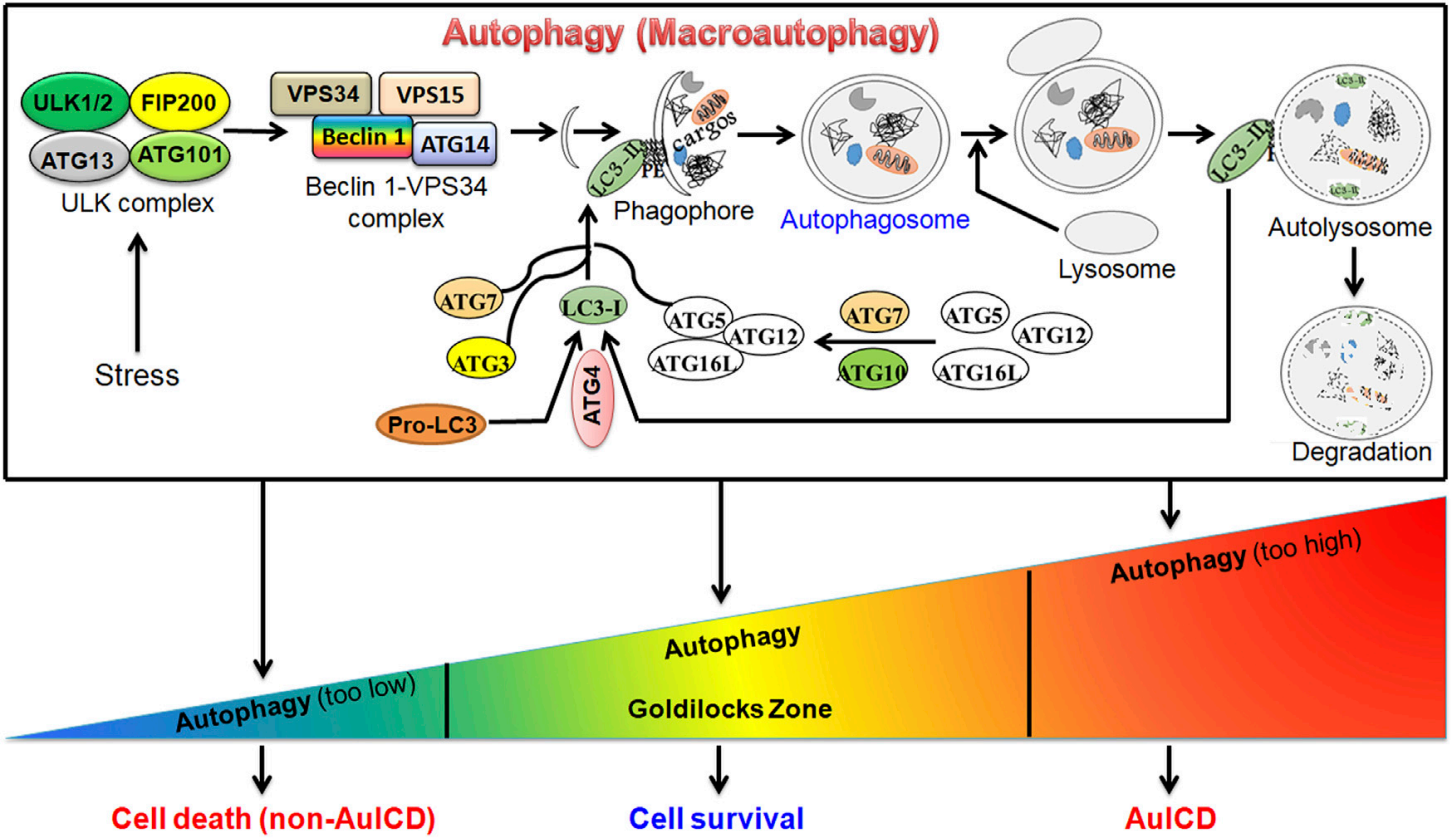
A graphic model for the canonical autophagy pathway and roles of autophagy in cell survival and death. Under stress (e.g., starvation), autophagy is induced. This is followed by the formation of autophagosome, a double-membrane vesicle, which fuses with the lysosome to form autolysosome to degrade cargo(s) by lysosomal enzymes. Autophagy can be at different levels. In the homeostatic “Goldilocks Zone” condition, autophagy promotes cell survival and prevents cell death. When it is deregulated, cell death can be induced via two mechanisms. First, when autophagy is highly downregulated, the accumulation of damaged cellular organelles, such as dysfunctional mitochondria, or other components triggers cell death. Second, when autophagy is highly upregulated, it degrades essential cellular compoents or organelles such as mitochondria leading to autophagy-induced cell death (AuICD).

Autophagy (macro-autophagy) is an evolutionarily conserved, intracellular “self-digestion” catabolic process characterized by the formation of double-membrane autophagosomes that sequester and enclose cytoplasmic materials (cargos) and fusion of autophagosome with lysosome for degradation (Figure 1). Autophagy is a stress-responsive mechanism that supports eukaryotic cells to survive harsh environments benefiting their growth. However, deregulated autophagy triggers cell death. A wide array of research studies support the crucial role of autophagy in a variety of diseases. Cell death instigates the pathogenesis of several diseases such as liver and heart disease while it prevents and treats cancer. Autophagy has dual roles in both preventing and inducing cell death by scavenging damaged and aged cell organelles and excessively degrading essential cellular organelles such as mitochondria, respectively. Understanding the roles and mechanisms of autophagy in cell death and survival during disease progression could provide a novel and effective strategy to prevent and treat disease. Autophagy is a dynamic process, which alters in a temporal fashion and depends on various factors including stress type, stress duration, and cell type. Thus, the level of autophagy does not remain constant, and it varies in a time and context-dependent manner. Therefore, unraveling the specific roles of autophagy in different types of diseases and different pathological conditions is warranted. The published studies on this topic have elucidated novel regulatory roles of autophagy in hepatocellular carcinoma, pancreatic cancer, glioblastoma, triple negative breast cancer, intervertebral disc degeneration, and liver disease. These studies will further our understanding of the crucial roles of autophagy in cell death and survival and trigger future related studies in other diseases.

## New Findings Reported in This Research Topic

It should be noted that a comprehensive inclusion of the current new findings on this research topic could not be possible due to the authors’ diverse choices in journals. We have collected five original research articles and one review article on this research topic, which provides a glimpse of the ongoing research in the area of autophagy regulation in cell death and survival in diseases.

More than 30 autophagy related (ATG) genes and their proteins are involved in the canonical autophagy pathway. In recent years, there is an emerging interest in investigating the roles of individual ATG proteins in disease progression and treatment. In this research topic, two studies have reported the roles of ATG4 proteins in cancer cell death and proliferation. In mammals, four isoforms of ATG4 (ATG4A, ATG4B, ATG4C, and ATG4D) exist. Chen et al. (Chen et al.) reported that the long non-coding RNA (lncRNA) colorectal neoplasia differentially expressed (CRNDE) promotes autophagy by stabilizing *ATG4B* mRNA to upregulate ATG4B protein. This is involved in promoting sorafenib-induced drug resistance in hepatocellular carcinoma (HCC) cells and tumors. In contrast, Nie et al. (Nie et al.) demonstrated that ATG4D-dependent mitophagy (mitochondria-selective autophagy) promotes PPARγ antagonist (drug T0070907)-induced apoptotic cell death and suppresses tumor growth of pancreatic cancer cells. Although the roles of ATG4B and ATG4D in drug resistance and tumor growth of the same type of cancer cells need to be investigated in future studies, the results from these two studies support that autophagy induction driven by different ATG proteins could have opposite effects on cancer drug resistance and tumor progression.

Similar to the pro-cell survival mechanism of ATG4B-dependent autophagy in HCC (Chen et al.), Eicosapentaenoic acid (EPA)-induced autophagy inhibits apoptosis, suppresses endoplasmic reticulum stress and the degradation of extracellular matrix in a rat model. Altogether, they alleviate intervertebral disc degeneration (IDD), which is a major cause of low back pain (Lin et al.). Similar to the report on the cell death-promoting effect of ATG4D-dependent autophagy (Nie et al.), Chen and Gibson (Chen and Gibson) demonstrated that knockout of the novel tumor suppressor TSSC4 (tumor suppressing subtransferable candidate 4) increases autophagy-induced cell death (AuICD) instigated by temozolomide (TMZ) in glioblastoma (GBM) cells.

Studies in recent years have suggested that ATG proteins can function independently of autophagy. Here, Rajak et al. (Rajak et al.) have comprehensively reviewed the current understanding of autophagy-dependent and -independent functions of hepatic ULK1 signaling in liver disease.

Finally, based on the DNA homologous recombination repair deficiency and bioinformatics analysis, Liao et al. (Liao et al.) published a model to predict anticancer drugs suitable for treating triple-negative breast cancer. As autophagy plays a critical role in DNA homologous recombination repair, understanding the roles of autophagy on anticancer activities of these predicted drugs may lead to new strategies to improve cancer treatment.

## Future Perspectives

The majority of this research topic included new original studies revealing pro-cell survival and pro-cell death roles of autophagy in disease progression and treatment, which supports the notion that the functions of autophagy are context-dependent. Based on the diseases studied in this research topic, novel therapeutic strategies can be utilized to target the pro-cell survival or pro-cell death roles of autophagy in various diseases. For example, the pro-cell survival mechanism of autophagy can be targeted to treat cancer cells. The levels of autophagy must be maintained in a “Goldilocks Zone” to maintain cellular homeostasis as suppressing or exacerbating autophagy could lead to cell death via autophagy-independent mechanisms or autophagy-induced cell death (AuICD). Driving autophagy out of the Goldilocks Zone in cancer cells has the potential to overcome drug resistance for cancer treatment (Figure 1).

As reported in this research topic, an emerging focus in the field of autophagy is to study the roles of individual ATG proteins in human diseases, where autophagy-independent functions of these proteins may play a role. ULK1, an ATG protein, has autophagy-independent roles in the liver, which needs further investigation in liver disease and potentially other metabolic diseases.
